# Health as a bridge to peace and trust in Myanmar: The 21^st^ Century Panglong Conference

**DOI:** 10.1186/s12992-017-0271-3

**Published:** 2017-06-28

**Authors:** Kun Tang, Yingxi Zhao

**Affiliations:** 1 Peking University Department of Global Health, #38 Xueyuan Road, Haidian, Beijing, China; 2 Peking University Institute for Medical Humanities, #38 Xueyuan Road, Haidian, Beijing, China

**Keywords:** Myanmar, Peace-building, Health system, Ethnic

## Abstract

The twenty-first Century Panglong Conference, proposed by Aung San Suu Kyi and her National League for Democracy, has concluded with unsatisfactory results. This five-day conference attracted global attention and wide endorsements from the United Nations and many other key stakeholders. The broad framework of the peace dialogue included various social and economic issues. However, the implication of the conference on the health system strengthening efforts in ethnic areas was largely unknown. Although a “convergence model” was proposed by organizations in Thai-Myanmar borders as a roadmap for integrations of the national and ethnic health systems years ago, the genuine bottlenecks beneath have not been addressed. This commentary discussed the Panglong Conference and its implication for the health systems in ethnic regions, as well as the bottlenecks of a “peace process” in health sector. It outlined a few key steps to achieve health system convergence between national and ethnic health systems, the outcome of which will not only improve the health status of the ethnic regions, but also help strengthen mutual trust and understanding among peoples, as a powerful bridge for peace.

**Fig. 1:**
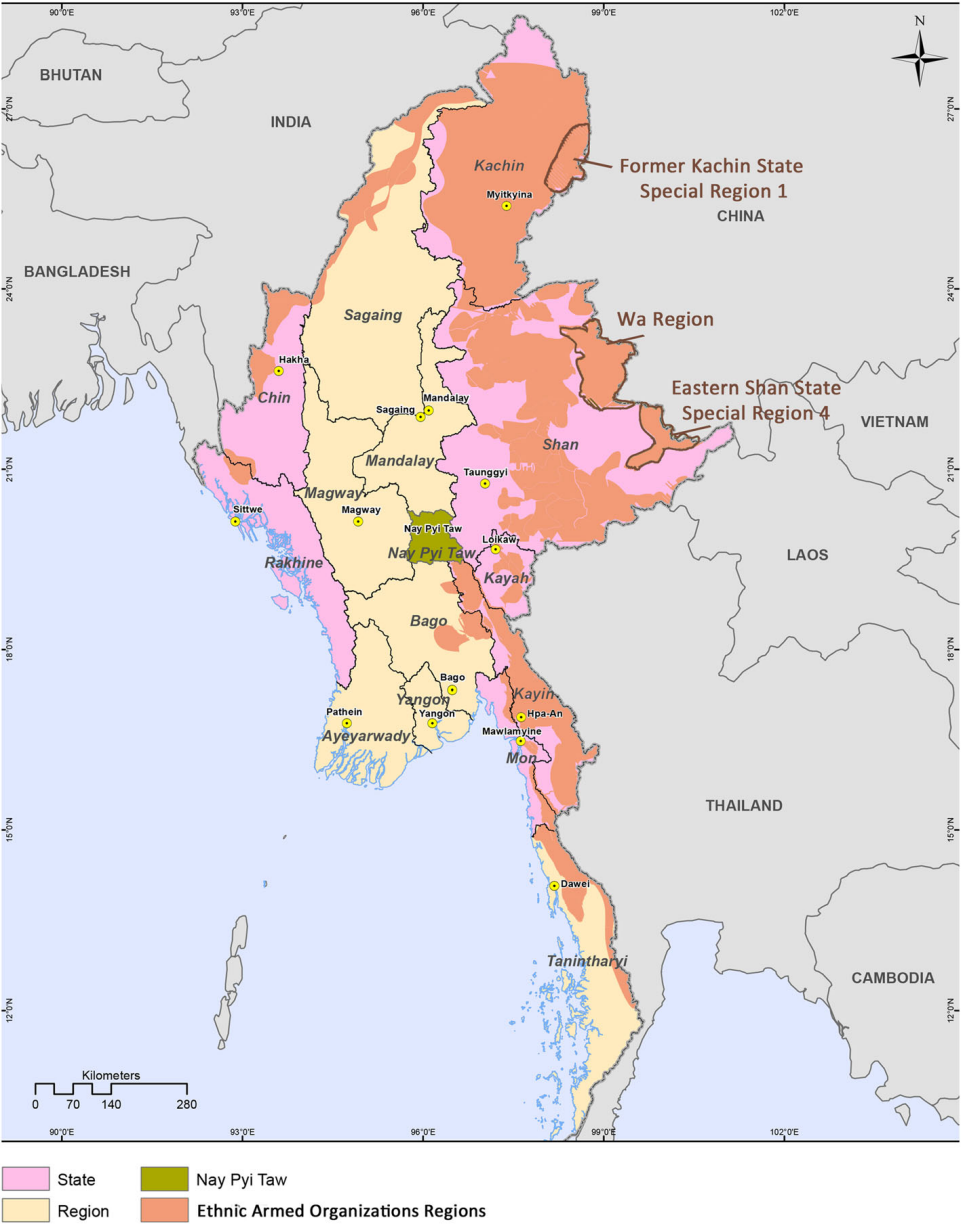
Ethnic Armed Organizations Controlled Areas in Myanmar

Almost 70 years after the Panglong Agreement, the Myanmar government, parliament, tatmadaw and 17 ethnic armed organizations (EAO) finally agreed to establish a political dialogue for their national peace and reconciliation—a cornerstone twenty-first Century Panglong Conference proposed by Aung San Suu Kyi and her National League for Democracy [1]. The Conference tries to tackle a comprehensive roster of political issues including a full national ceasefire, but a failed “all-inclusiveness” and the departure of one EAO from the conference [2] has shown a fragile trust between the two sides. Moreover, a recent airstrike near Muse and Kutkai townships in Shan State that resulted in ten death and over 3000 people fled across the border into China also reflected the failed negotiation [3]. Within this general dissatisfaction, one specific concern is the lack of attention to health in the peace dialogues. Although health has been shown to play a constructive role in peace-building and national reconciliation, it is perhaps one of the lowest priorities in the initial stage of the peace talks.

Myanmar’s health statistics are notoriously weak. While Myanmar’s infant and maternal mortality is toward the lower range in the Greater Mekong Sub-region, the health status in ethnic conflict areas is more inferior. Infant mortality and under five mortality rate in the Myanmar-Thailand border area was estimated at 77 and 139 per 1000 births [4], much higher than any other regions of the country. A recent malaria survey along the Myanmar-China border suggested an incidence of 8.7 per thousand [5], almost twice Myanmar’s national average [6]. Health disparities in ethnic regions are a major result of the prolonged armed conflicts and a lack of economic development, which leads to a failure to develop or the collapse of local health systems.

Health as a bridge for peace has already seen success in UNICEF’s ceasefire for immunization in El Salvador [7] and the WHO’s decentralized cooperation in conflicted Bosnia and Herzegovina [8]. In these scenarios, addressing disparities in health and gaps in health services served to mitigate conflict because health issues transcend political, economic, social and ethnic divisions among peoples and provide a nexus for dialogue at multiple levels [9]. It directly addressed social inequities, injustice and set a space for dialogue of common interests. In the case of Myanmar, many see health as a crucial instrument for trust-building, as it is one of the few social domains where the central government and EAOs can agree to collaborate despite their political dissidence. Decades of isolation and militarization leaves the ethnic areas with feeble health system, thus integrating health services of two sides (known as the “health convergence model”, which was raised by the ethnic groups on the Thai-Myanmar border area) [4] may create a positive interaction between Myanmar’s national and ethnic health systems, with the hopes that it will narrow the health gap and also promote mutual understanding and trust between the two peoples. Translating these hopes, however, will have to grapple successfully with many barriers.

At the start, a critical issue is the exclusivity between the central government and EAOs and their divergent priorities for health system development. For ethnic authorities, building a stronger “stand-alone” health system is desirable and consistent with their political pursuit of local autonomy under federalism. In contrast, the central government sees replacing local health systems with a unified national system as a path towards national unification. Neither position, however, would bridge the health gaps. On one hand, the current ethnic health systems are often too broken to revive, as they have long suffered from minimal funding support, severe shortage of human resource, outdated technologies and low quality of care. Many public health functions are undertaken by international NGOs, without which many services would collapse. On the other hand, a complete replacement would leave a vacuum in local health service delivery that could persist for many years. A case in point is Kachin Special Region One (Fig. 1), where 7 years after ethnic authority handed off all public services to the central government, its health status remains one of the poorest among all the ethnic regions, with a HIV prevalence of 11% among general population [10], a child immunization coverage of only 29% [11] and less than half of its clinics fully functioning.

As a transitional scenario, a model with both systems co-existing and complementing each other, which has been observed in Eastern Shan State Special Region 4 and Wa Region (Fig. 1), seems promising. In the case of these two regions, the Ministry of Health and Sports (MoHS) and the EAO health authorities oversees the health sector in parallel. The national and local health systems both provide basic services in the ethnic regions and jointly conduct health planning and regulation under the coordination of international donors [4]. Extendng the national health system to these ethnic regions has helped to shore up weaknesses in the ethnic health system and achieved wider health coverage is in a relatively short period of time, though a long-term improvement in people-to-people relations requires that down-to-earth health services made available to ethnic people through coordinated efforts, including free flow of essential medicines, information, services, infrastructures and capacities between the two systems.

The co-existence model is a positive first step. For a universal convergence, some fundamental issues need to be addressed. First, the process must ensure ethnic people’s full participation in decision-making of their local health development. The Myanmar central government should decentralize its health planning as a way to address ethnic disparities and equitable access to healthcare. Cultural differences and language barriers should also be properly handled as a breaker for the long-hold distrust between peoples, as the lack of inclusiveness and cultural identify are considered the major causes for the conflict in the ethnic regions. Second, the key focus of convergence should be alignment on with national standards, guidelines and trainings, while enhancing local capacities. Instead of simply transferring medical personnel from national system to local systems, building a localized ethnic health workforce is the only sustainable solution. Finally and above all, a strong commitment to place health high on the peace-agenda and strive for improvement in health, along with open-mindedness and a degree of flexibility of both sides, is important to build trust and cooperation for a real health convergence.

## Conclusion

Needless to say, attaining peace is essential for the health of millions living in the ethnic regions. After decades of civil war, none of the critical political complexities can be resolved unless mutual trust, respect and genuine commitment to reconciliation prevails the twenty-first Century Panglong peace negotiations. Promoting health convergence is not only a goal in its own right, but as a means to strengthen trust and mutual understanding between peoples, can also be a powerful bridge for peace.
